# Identification of the active substances and mechanisms of ginger for the treatment of colon cancer based on network pharmacology and molecular docking

**DOI:** 10.1186/s13040-020-00232-9

**Published:** 2021-01-11

**Authors:** Meng-Meng Zhang, Dan Wang, Feng Lu, Rong Zhao, Xun Ye, Lin He, Li Ai, Chun-Jie Wu

**Affiliations:** 1 School of Pharmacy, Chengdu University of Traditional Chinese Medicine, No.1166 Liutai Avenue, Chengdu, 611137 P. R. China; 2 School of Ethnic Medicine, Chengdu University of Traditional Chinese Medicine, No.1166 Liutai Avenue, Chengdu, 611137 P. R. China

**Keywords:** Ginger, Colon cancer, GO enrichment, KEGG enrichment, Molecular docking, 1-Monolinolein

## Abstract

**Background and objective:**

Colon cancer is occurring at an increasing rate and ginger (*Zingiber officinale*)*,* as a commonly used herbal medicine, has been suggested as a potential agent for colon cancer. This study was aimed to identify the bioactive components and potential mechanisms of ginger for colon cancer prevention by an integrated network pharmacology approach.

**Methods:**

The putative ingredients of ginger and its related targets were discerned from the TCMSP  and Swiss target prediction database. After that, the targets interacting with colon cancer were collected using Genecards, OMIM, and Drugbank databases. KEGG pathway and GO enrichment analysis were performed to explore the signaling pathways related to ginger for colon cancer treatments. The PPI and compound-target-disease networks were constructed using Cytoscape 3.8.1. Finally, Discovery studio software was employed to confirm the key genes and active components from ginger.

**Results:**

Six potential active compounds, 285 interacting targets in addition to 1356 disease-related targets were collected, of which 118 intersection targets were obtained. A total of 34 key targets including PIK3CA, SRC, and TP53 were identified through PPI network analysis. These targets were mainly focused on the biological processes of phosphatidylinositol 3-kinase signaling, cellular response to oxidative stress, and cellular response to peptide hormone stimulus. The KEGG enrichment manifested that three signaling pathways were closely related to colon cancer prevention of ginger, cancer, endocrine resistance, and hepatitis B pathways. TP53, HSP90AA1, and JAK2 were viewed as the most important genes, which were validated by molecular docking simulation.

**Conclusion:**

This study demonstrated that ginger produced preventive effects against colon cancer by regulating multi-targets and multi-pathways with multi-components. And, the combined data provide novel insight for ginger compounds developed as new drug for anti-colon cancer.

## Introduction

With the rapid development of human society and the improvement of life standard, cancer as a growing threat, is the second leading noncommunicable disease of death globally next only to cardiovascular disease [1]. Colorectal cancer is a broad term that includes patients with colon cancer and rectal cancer [2]. In 2018, there are 1,849,518 new cases of colorectum cancer worldwide and 880,792 deaths (https://gco.iarc.fr/). Currently, increasing therapeutic approaches for colon cancer treatment are available, not only the conventional treatments of surgical resection and radiotherapy but also new interventional therapies like drug therapy. However, high costs of molecular targeted therapy and chemotherapy-induced adverse effects such as nausea, vomiting, and digestive tract irritation reaction always leads to treatment drop out.

Historically medicinal botanicals, as one part of complementary medicine in the USA, have provided an important resource for discovering anticancer drug agents, and more than half of currently available drugs are related to them [3]. Ginger (*Zingiber officinale)*, belong to Zingiberaceae family, has long been used as a traditional Chinese medicine (TCM), which mainly distributed in India, China, and Nigeria [4]. Ginger rhizome as the major medicinal part contains pungent phenolic compounds, volatile oil, diarylheptanoids, and phenylalkanoids, leading to a wide range of pharmacological effects [5, 6]. Furthermore, 6-gingerol and 6-shogaol, the representative components derived from ginger, has been reported to preventing cell proliferation against colon cancer, such as Colon-26 tumors [7, 8]. However, it is unclear whether other ginger components possess the anticancer activity.

While moving ahead with computer technology, network pharmacology incorporating a series of disciplines and techniques has emerged. Network pharmacology has been successfully introduced to reveal the therapeutic mechanisms of TCM, as it in accordance with the connotation of holistic strategy of multi-components, multi-targets and multi-pathways [9–11].

**Fig. 1:**
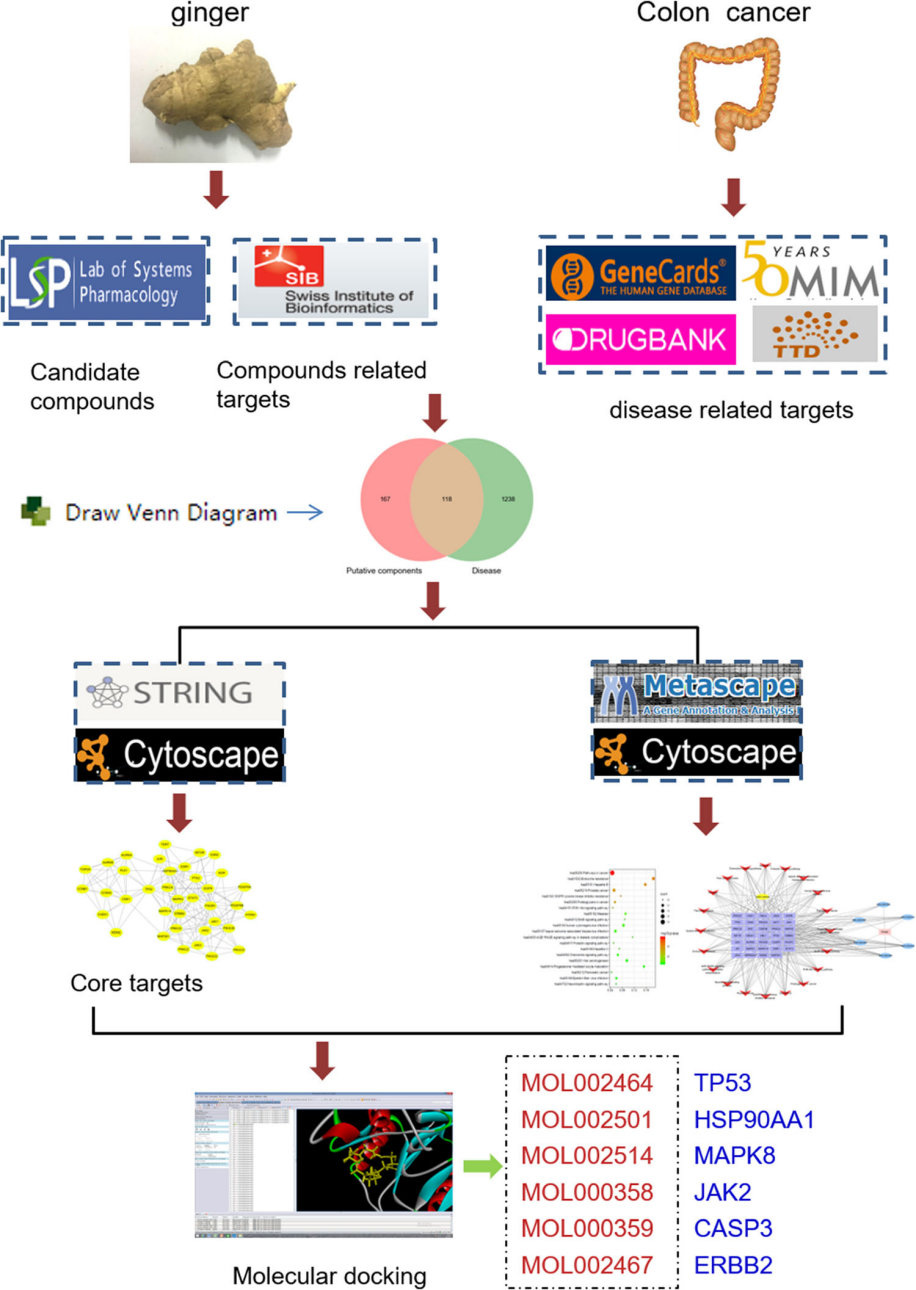
The detailed flowchart of the study design

In this study, network pharmacology was conducted to establish the components-targets-pathways-disease network to investigate the potential mechanisms of ginger in colon cancer prevention. The detailed flowchart of the study design was shown in Fig. 1. Firstly, the candidate compounds and intersection genes for colon cancer were collected. Then, the protein-protein interaction (PPI) and components-targets-pathways-disease network were constructed. In addition, Gene Ontology (GO) and Kyoto Encyclopedia of Genes and Genomes (KEGG) analysis were performed. Finally, the potential bioactive components and core targets were further validated by the molecular docking simulation.

## Materials and methods

### Active components and corresponding target collection

Traditional Chinese medicine systems pharmacology (TCMSP, http://tcmspw.com/tcmsp.php) database is a platform containing many compounds from herbal medicine, related protein targets as well as its pharmacokinetic properties [12]. It was used to collect the potential active components of ginger, by filtering the metrics of oral bioavailability (OB) ≥ 30% and drug-likeness (DL) ≥ 0.18 [13]. DL is a qualitative property of chemicals and is widely used in the early stages of drug discovery. OB refers to the relative amount and rate at which an oral drug is absorbed by the body into the systemic circulation. OB and DL, calculated by machine learning methods or Tanimoto coefficient, are commonly used for filtering out compounds that are unlikely to be drugs. Subsequently, protein targets interacting with those potential active compounds were predicted through TCMSP and Swiss Target Prediction (http://www.swisstargetprediction.ch/) databases [14].

### Acquisition of disease-associated targets and candidate genes

Keywords such as “colon cancer” or “colon tumor” were used to search the known targets related to the pathogenesis of colon cancer from three major databases, GeneCards (https://www.genecards.org) [15], OMIM (https://omim.org/) [16], and Drugbank database (https://www.drugbank.ca/). To reduce the false positives, only the curated targets that directly associated with the disease were included, and the repetitive targets were removed. Then, the protein targets related to disease and ginger compounds were both imported into Uniprot database (https://www.uniprot.org/) to acquire its related gene name, gene ID and functions, respectively. The obtained genes were both inputted to draw Venn diagram (http://bioinformatics.psb.ugent.be/webtools/Venn/), and the intersection genes were collected as candidate genes.

### Protein-protein interaction (PPI) network

Based on the candidate genes, a PPI network was constructed by importing the candidate genes to the Search Tool for the Retrieval of Interacting Genes (STRING, https://string-db.org/) database, with a highest confidence of 0.9 [17]. Cytoscape software, version 3.8.1 [18] was used to visualize the PPI network. Then, three topological features, “degree”, “betweenness”, and “closeness” were calculated to identify the key genes. “Degree” parameter represents the number of edges associated with a node. “Betweenness” indicates the number of shortest paths between pairs of nodes, and “closeness” describes the inverse of the number of distances.

### GO and KEGG pathway enrichment analysis

The Gene Ontology (GO) and Kyoto Encyclopedia of Genes and Genomes (KEGG) enrichment were performed by the Database for Metascape (https://metascape.org). GO functionally annotates key genes into three main terms, including cellular components (CCs), molecular functions (MFs), and biological processes (BPs). Besides, Cytoscape ClueGO plugin was employed to further analyze BPs enrichment. KEGG enrichment analysis unveils the possible biological process with key genes. In addition, the bubble chart of GO and KEGG enrichment analysis were performed on the bioinformatics platform (http://www.bioinformatics.com.cn/).

### Construction of drug-components-disease-targets-pathways network

To characterize the therapeutic mechanisms of ginger for colon cancer, a network of drug-components-disease-targets-pathways was constructed using Cytoscape software, version 3.8.1 [18]. In the network, the nodes with different colors and shapes represent the drug, components, disease, target genes, or disease related pathways, respectively, and an “edge” is an association between the nodes.

### Molecular docking simulation

A total of 8 key genes which have good correlation with other genes, active components, and pathways including SRC, PIK3R1, TP53, HSP90AA1, MAPK8, JAK2, CASP3, and ERBB2 were included in the molecular docking simulation. The PDB-ID of these target genes were accessed from Protein Data Bank (PDB) database. In brief, Discovery studio software (version 4.5.0, Biovea Inc., Omaha, NE, USA) was employed, and the screened active components were prepared using “Prepare Ligands” module to obtain an effective three-dismensional conformation. After removing crystallographic water molecules, the “Prepare Protein” module was used to remove the polyconformation of target protein and to supplement the incomplete amino acid residues. Subsequently, molecular docking was performed in “LibDock” module, and LibDockScore was required to evaluate affinity of the target proteins and active components. The LibDockScore of the target protein and its corresponding prototype ligand was viewed as the threshold, and the components with higher scores were regarded as the active ones for interacting with this protein.

## Results

### Ginger components and candidate genes associated with colon cancer

After searching, filtering, and removal of the duplicates, 6 putative components (MOL002464, MOL002501, MOL002514, MOL000358, MOL000359, and MOL002467) with OB ≥ 30% and DL ≥ 0.18 were selected from TCMSP database (listed in Table 1). Besides, the representative component 6-gingerol with high contents and multiple pharmacological activitieswas also recruited in this study, although the DL value is 0.16.

**Table 1:**
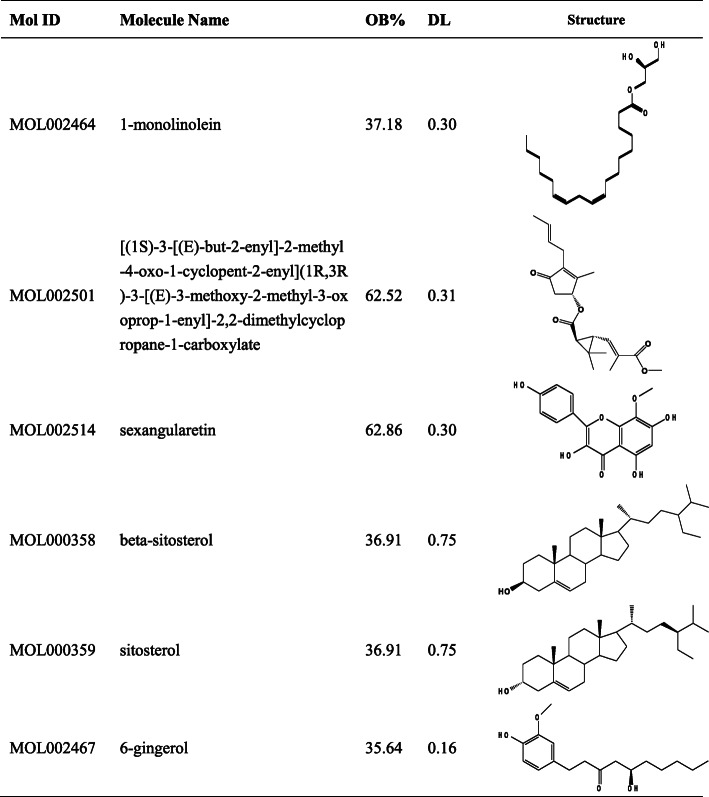
The active compounds and their properties and structures

**Fig. 2:**
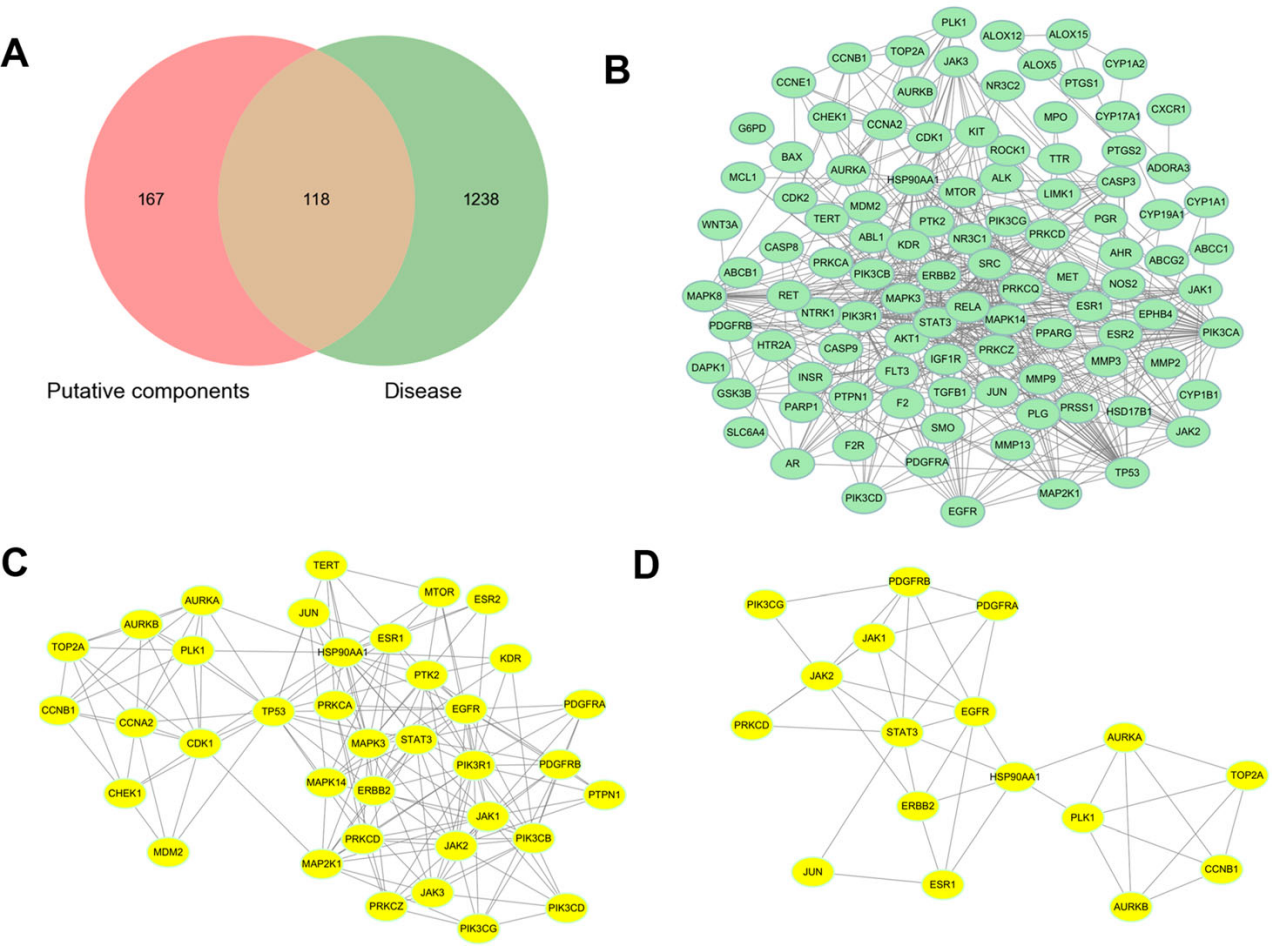
The representative pictures of Venn diagram and protein-protein network. **a** 118 intersection genes; **b** a complete protein-protein network; **c** one protein-protein cluster with 37 nodes and 179 edges; **d** one protein-protein cluster with 17 nodes and 42 edges

Meanwhile, 285 target genes in total interacting with those putative components were collected, among which 251 were obtained from the Swiss Target Prediction and 34 from TCMSP database. In addition, there remained 1356 colon cancer-associated genes, in which 1165 genes were from the GeneCards database, 156 from the OMIM, and 35 from the Drugbank. Finally, a total of 118 intersection genes, also the candidate genes, were collected for further mechanisms study of ginger on treatment of colon cancer (Fig. 2a).

### PPI network analysis

The 118 candidate genes were connected to establish an initial PPI network using Cytoscape 3.8.1 that included 104 nodes and 517 edges, and 14 isolated targets genes were removed (Fig. 2b). In addition, the top two protein-protein interacting clusters were constructed (Fig. 2c and d). Genes PIK3R1, CCNA2, and TP53 were as the core targets for one cluster (37 nodes and 179 edges), while another cluster (17 nodes and 42 edges) with STAT 3 and JAK2 as the core targets. Based on the analysis of this network, only the genes with higher values of “degree”, “betweenness” and “closeness” (above the median value) were collected as the key targets of ginger for colon cancer. Ultimately, 34 key targets, with PIK3CA, SRC, and TP53 as the top ones, were collected for pathway enrichment analysis (Table 2).

**Table 2:** The major targets of ginger for colon cancer treatment and the topological parameters

Uniprot ID	Gene symbol	Target name	Degree	Betweenness	Closeness
P42336	PIK3CA	Phosphatidylinositol 4,5-bisphosphate 3-kinase catalytic subunit alpha isoform	42	0.187322	0.572254
P12931	SRC	Proto-oncogene tyrosine-protein kinase	38	0.107991	0.568966
P27986	PIK3R1	Phosphatidylinositol 3-kinase regulatory subunit alpha	38	0.106891	0.568966
P04637	TP53	Cellular tumor antigen p53	35	0.103695	0.546961
P27361	MAPK3	Mitogen-activated protein kinase 3	33	0.084312	0.543956
P40763	STAT3	Signal transducer and activator of transcription 3	31	0.080670	0.535135
P31749	AKT1	RAC-alpha serine/threonine-protein kinase	29	0.068649	0.532258
P07900	HSP90AA1	Heat shock protein HSP 90-alpha	27	0.066522	0.529412
P45983	MAPK8	Mitogen-activated protein kinase 8	23	0.065784	0.502538
O60674	JAK2	Tyrosine-protein kinase JAK2	20	0.056021	0.497487
P42338	PIK3CB	Phosphatidylinositol 4,5-bisphosphate 3-kinase catalytic subunit beta isoform	19	0.036367	0.492537
P00533	EGFR	Epidermal growth factor receptor	19	0.028652	0.485294
Q05513	PRKCZ	Protein kinase C zeta type	18	0.021649	0.480583
Q05655	PRKCD	Protein kinase C delta type	18	0.021286	0.478261
P23458	JAK1	Tyrosine-protein kinase JAK1	18	0.017349	0.478261
P03372	ESR1	Estrogen receptor	18	0.016778	0.473684
Q04206	RELA	Transcription factor p65	17	0.014046	0.469194
Q16539	MAPK14	Mitogen-activated protein kinase 14	17	0.011251	0.469194
Q05397	PTK2	Focal adhesion kinase 1	15	0.009972	0.464789
Q02750	MAP 2 K1	Dual specificity mitogen-activated protein kinase kinase 1	15	0.009205	0.464789
P04626	ERBB2	Receptor tyrosine-protein kinase erbB-2	15	0.008965	0.464789
P42574	CASP3	Caspase-3	15	0.008693	0.462617
P04150	NR3C1	Glucocorticoid receptor	14	0.00863	0.460465
P05412	JUN	Transcription factor AP-1	14	0.008501	0.458333
P52333	JAK3	Tyrosine-protein kinase JAK3	14	0.008096	0.450000
P06493	CDK1	Cyclin-dependent kinase 1	14	0.007042	0.450000
P17252	PRKCA	Protein kinase C alpha type	13	0.006885	0.447964
P10275	AR	Androgen receptor	13	0.005944	0.445946
P08069	IGF1R	Insulin-like growth factor I receptor	11	0.005841	0.443946
O14965	AURKA	Aurora kinase A	11	0.005668	0.440000
P00519	ABL1	Tyrosine-protein kinase ABL1	11	0.005616	0.432314
Q00987	MDM2	E3 ubiquitin-protein ligase Mdm2	10	0.00552	0.415966
P24941	CDK2	Cyclin-dependent kinase 2	10	0.004538	0.415966
P49841	GSK3B	Glycogen synthase kinase-3 beta	8	0.004458	0.415966

### GO enrichment analysis

**Fig. 3:**
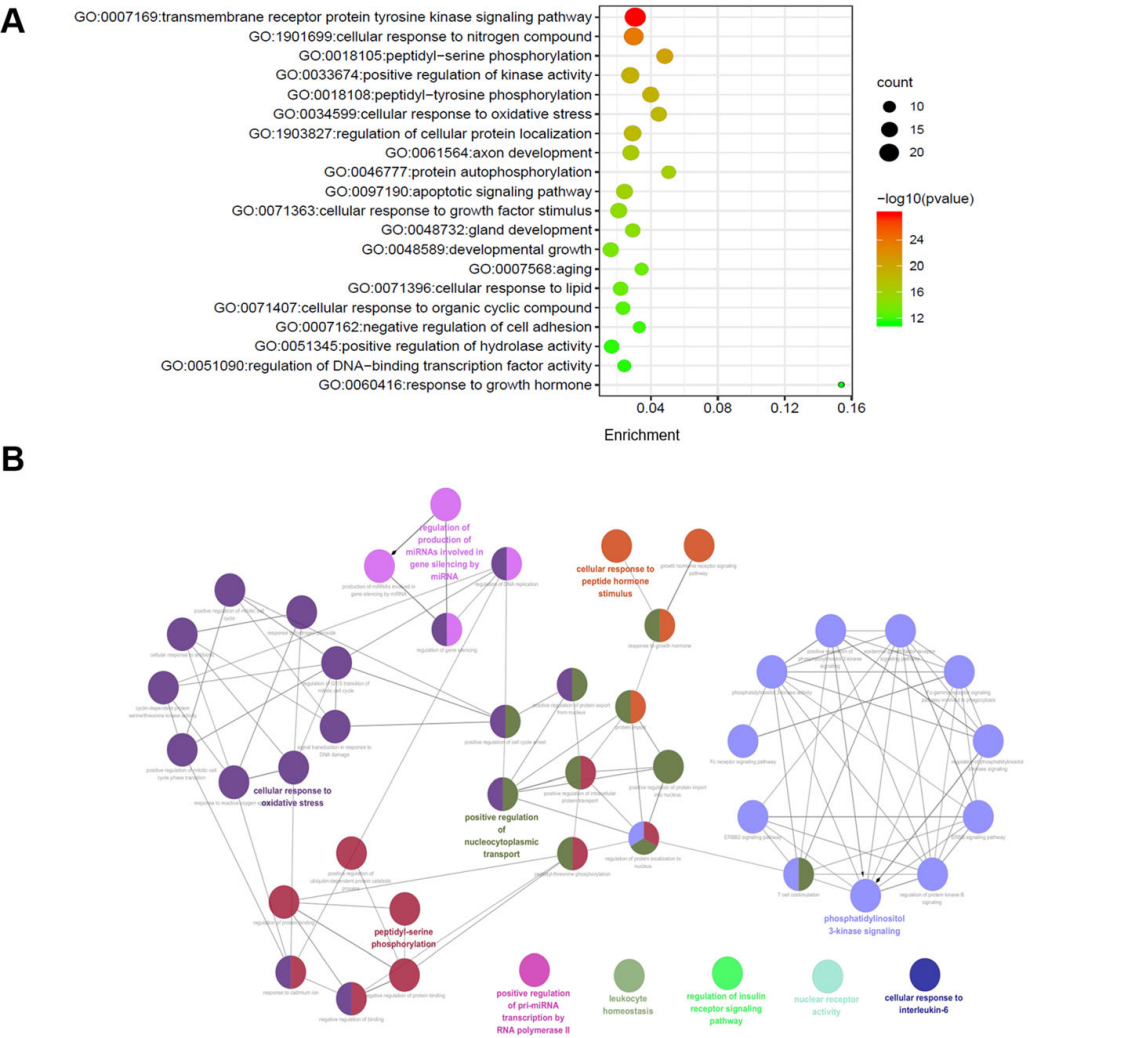
Enrichment analysis on BPs of 34 key targets. **a** The bubble chart; **b** the visualization analysis of BPs

To investigate the biological function of target genes of ginger for colon cancer, the biological process was obtained by the Metascape database. As shown in Fig. 3a, 20 markedly enriched BPs terms were shown (*p* < 0.01), with transmembrane receptor protein tyrosine kinase GO: 0007169, cellular response to nitrogen compound GO: 1901699, and peptidyl-serine phosphorylation GO: 0018105 as the top ones. The size of the dot in bubble chart indicates the number of target genes in the corresponding function pathway, and the enrichment expresses the ratio of the number of target genes belonging to all the annotated genes located in the pathway. Besides, further analysis of the network was performed using Cytoscape plugin ClueGO, and we found that these BPs terms were mainly associated with the phosphatidylinositol 3-kinase signaling, cellular response to oxidative stress, cellular response to peptide hormone stimulus, and peptidyl-serine phosphorylation (Fig. 3b).

For CCs, the items with significant enrichment were in perinuclear region of cytoplasm GO: 0048471, transferase complex, transferring phosphorus-containing groups GO: 0061695, and microtubule organizing center GO: 0005815; and for MFs in phosphotransferase activity, alcohol group receptor GO: 0016773, kinase binding GO: 0019900, and protein tyrosine kinase activity GO: 0004713.

### KEGG pathway enrichment analysis

**Fig. 4:**
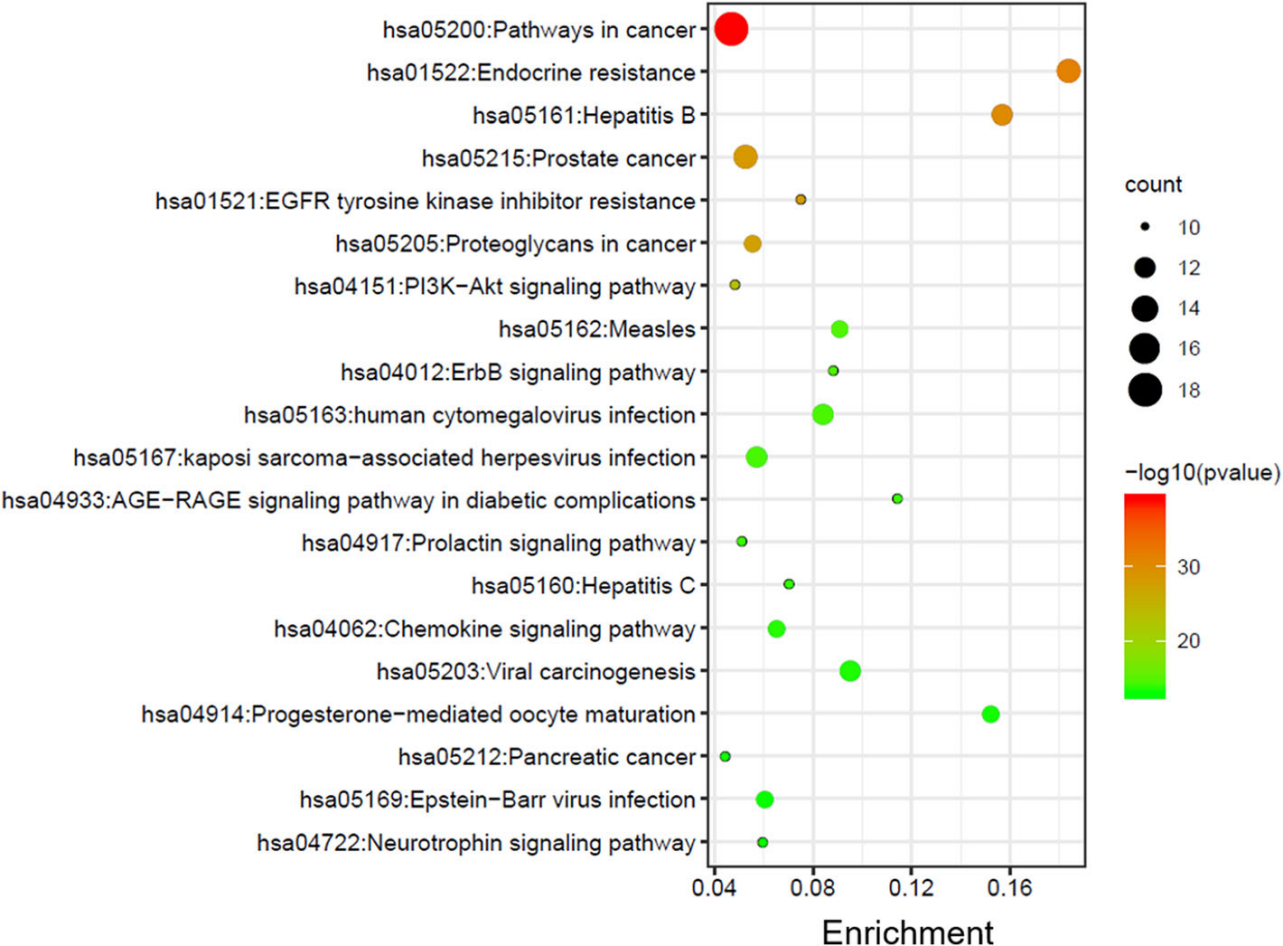
The bubble chart of KEGG enrichment based on 34 key targets

The KEGG enrichment showed how ginger acts on the pathway, thereby playing a therapeutic role in colon cancer. Here, based on the 34 core target genes, 20 significant signaling pathways (Fig. 4) with *p* < 0.01 were picked out for further analysis, including pathways in cancer (hsa05200), endocrine resistance (hsa01522), EGFR tyrosine kinase inhibitor resistance (hsa01521), and PI3K-Akt signaling pathway (hsa04151) as the top ones. Among these pathways, pathways in cancer was identified as a set of important key pathway with the most target enrichment and lowest *p* value.

### Drug-components-targets-pathways-disese network

**Fig. 5:**
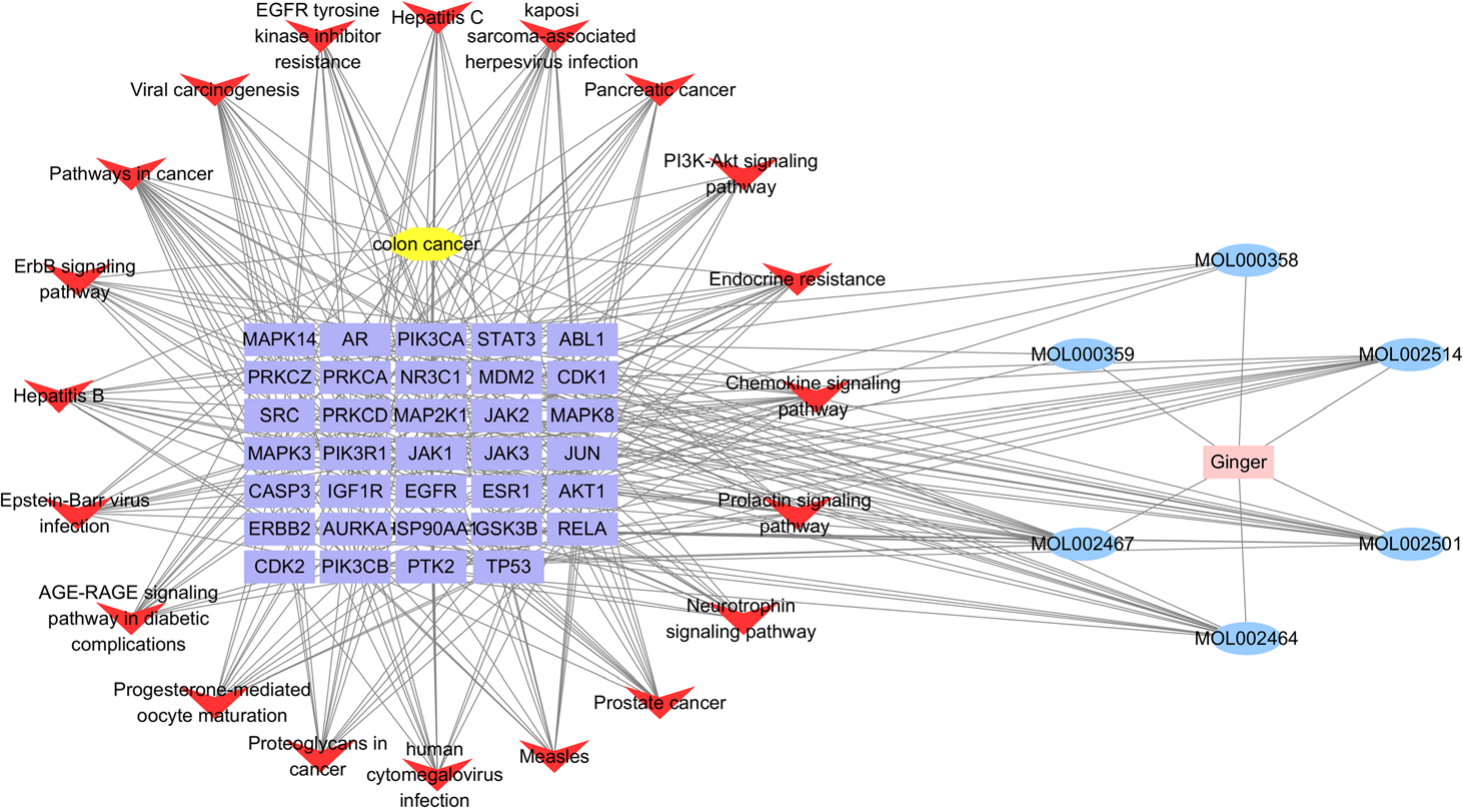
The drug-components-targets-pathways-disease network

The drug-components-targets-pathways-disease network was shown in Fig. 5, which included 62 nodes (6 compounds, 34 targets, and 20 pathways) and 311 edges. The pink rectangle node is the Chinese herbal medicine ginger; yellow nodes is colon cancer, V nodes is 20 significant signaling pathway; purple rectangle nodes are 34 key targets; blue ellipse node represents 6 potential active components, while lines represent the interactions between them. According to the network analysis, multiple components from ginger acts on at least one target genes, and 6-gingerol was regarded as the most effective compound that interacts with 17 target genes. Besides, most of target genes were regulated by at least 2 active components, and at least 10 genes potentially involved in each pathway related to colon cancer. This network analysis indicated the characteristics of multiple components and multiple targets of ginger in the treatment of colon cancer.

### Molecular docking results and analysis

**Fig. 6:**
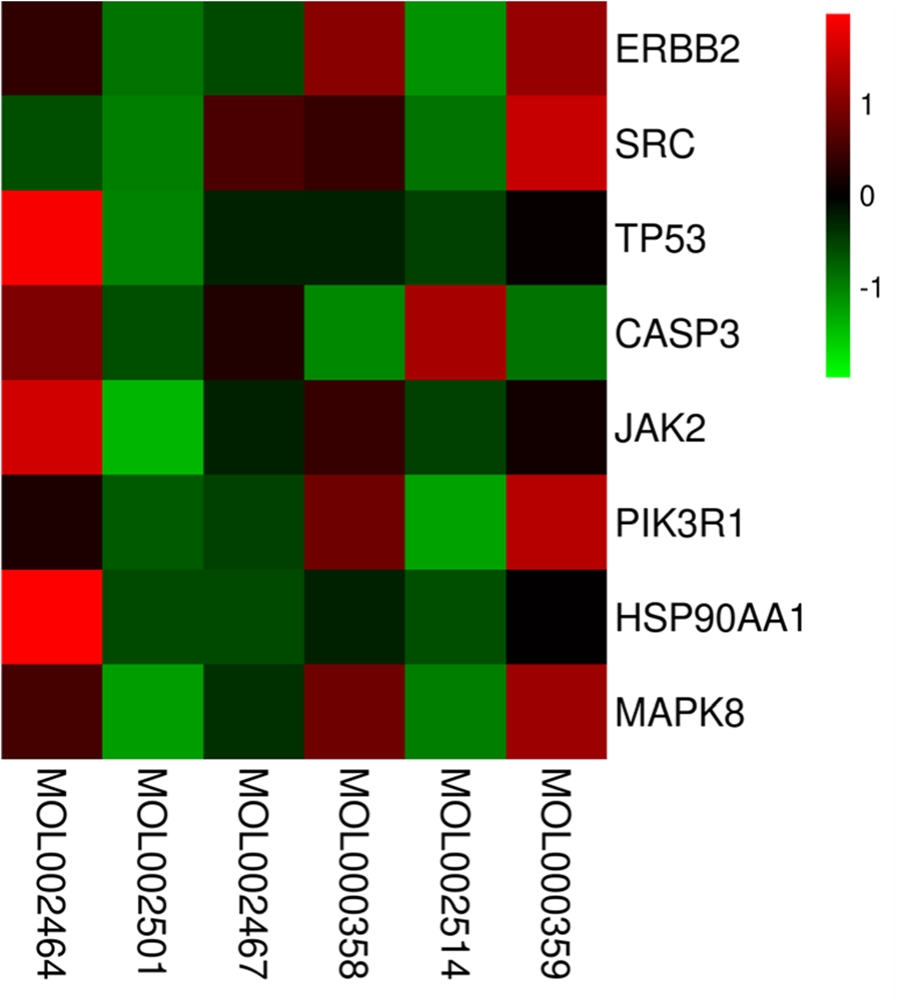
The heat map of the docking score

**Fig. 7:**
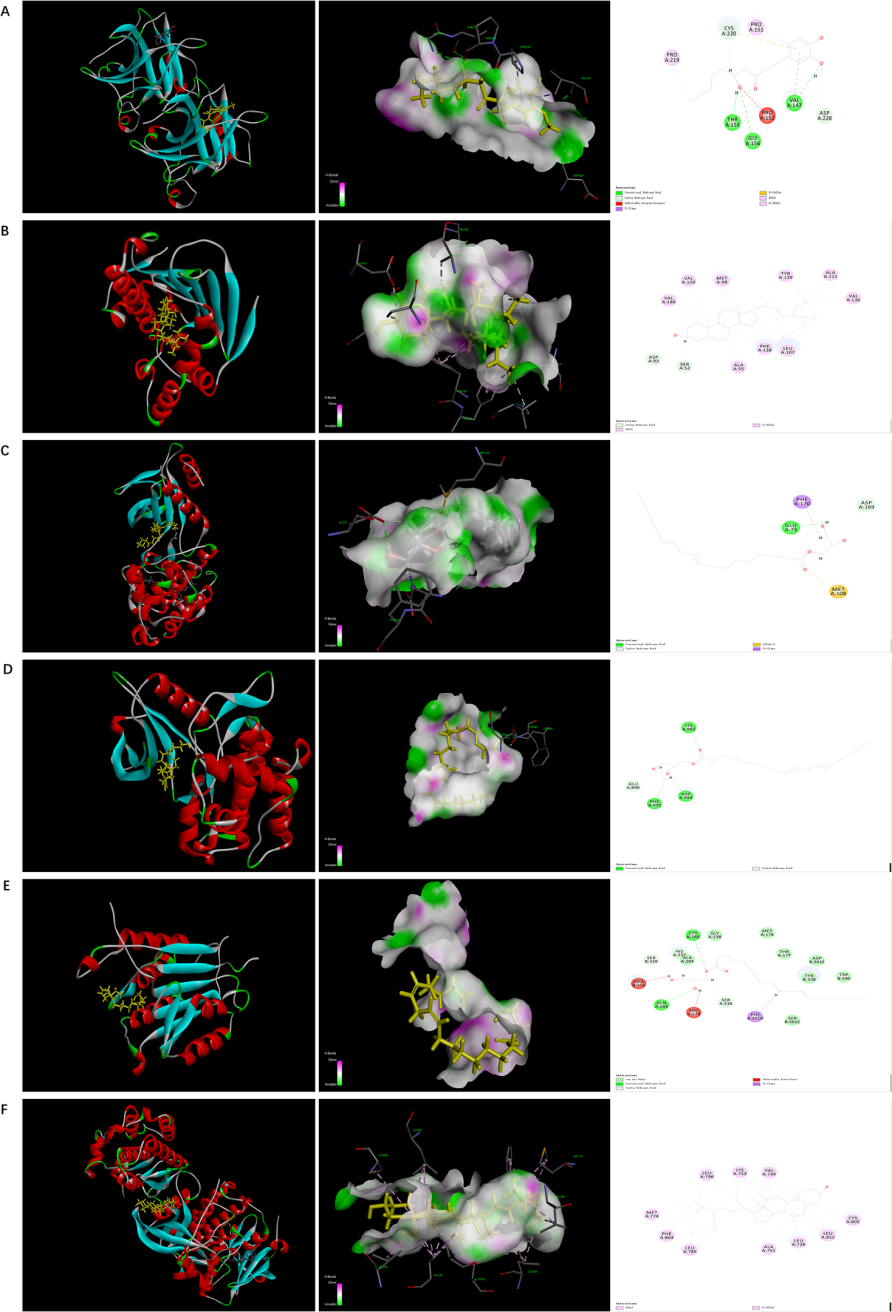
The represented results for the action mode of active compounds with 5 targets protein using molecular docking. **a** Action mode of 6-gingerol (MOL002467) with target TP53 (PDB ID: 5O1F); **b** Action mode of sitosterol (MOL000359) with target HSP90AA1 (PDB ID: 4BQG); **c**-**e** Action mode of 1-monolinolein (MOL002464) with target MAPK8 (PDB ID: 4E73), JAK2 (PDB ID: 3KCK), and CASP3 (PDB ID: 1RE1), respectively; **f** Action mode of beta-sitosterol (MOL000358) with target ERBB2

A total of 8 target genes which showed strong interactions with other targets, pathway and potential components were selected to binding with the 6 putative components. The binding affinities of the testing components, indicating as LibDock Scores, were compared to the original ligands of target protein. As LibDock Scores displayed in Table 3 and heat map of the docking score exhibited in Fig. 6, all the testing components had strong interactions than the prototype ligands, or similar effects to the ligands, with TP53, HSP90AA1 and JAK2. Especially for compound 1-monolinolein (MOL002464) showed good binding affinities with all the tested targets, except for SRC. In addition, 6-gingerol also showed a strong interaction with targets JAK2 and CASP3, while beta-sitosterol (MOL000358) and sitosterol (MOL000359) with ERBB2. These findings revealed that the six components including MOL002464, MOL002501, MOL002467, MOL000358, MOL002514, and MOL000359 were forecasted as the active components of ginger for colon cancer and TP53, HSP90AA1, and JAK2 were the major target for reaching this effect. The representative molecular docking results of the major targets and active components of ginger were exhibited in Fig. 7.

**Table 3:** The LibDock Scores of 8 core targets and their interacting compounds

Target	PDB ID	Components	LibDock Score
SRC	2BDF	Ligand 1	134.987
		MOL002464	125.359
		MOL002501	112.922
		MOL002467	115.549
		MOL000358	116.823
		MOL002514	106.056
		MOL000359	116.823
PIK3R1	4ZOP	Ligand 2	124.433
		MOL002464	127.529
		MOL002501	118.423
		MOL002467	112.848
		MOL000358	122.996
		MOL002514	107.208
		MOL000359	122.996
TP53	5O1F	Ligand 3	104.359
		MOL002464	146.759
		MOL002501	127.538
		MOL002467	128.495
		MOL000358	129.767
		MOL002514	125.236
		MOL000359	133.846
HSP90AA1	4BQG	Ligand 4	93.3973
		MOL002464	137.279
		MOL002501	130.015
		MOL002467	123.039
		MOL000358	124.673
		MOL002514	117.016
		MOL000359	124.673
MAPK8	4E73	Ligand 5	118.446
		MOL002464	126.778
		MOL002501	105.652
		MOL002467	99.7431
		MOL000358	110.918
		MOL002514	95.7262
		MOL000359	110.918
JAK2	3KCK	Ligand 6	102.423
		MOL002464	116.567
		MOL002501	105.396
		MOL002467	104.779
		MOL000358	109.404
		MOL002514	106.601
		MOL000359	109.106
CASP3	1RE1	Ligand 7	96.0197
		MOL002464	115.813
		MOL002501	100.826
		MOL002467	105.848
		MOL000358	82.4267
		MOL002514	104.926
		MOL000359	84.5633
ERBB2	3PPO	Ligand 8	120.872
		MOL002464	148.403
		MOL002501	109.008
		MOL002467	94.1141
		MOL000358	139.536
		MOL002514	91.1141
		MOL000359	139.034

## Discussion

Cancer is a worldwide problem, especially for the prevention of postoperative spread. Chinese herbal medicine, as the characteristics of safety and minimal side effects, are increasingly used as cancer-preventive agents for patients who prefer a healthy treatment after tumor resection. Recently, the network pharmacology has become an advanced method to analyze the complex mechanisms and collect active ingredients of Chinese herbs or TCM formula. This study indicated that ginger possess the effect of preventing colon cancer by regulating multi-targets with multi-components.

Specially, six ingredients of ginger were screened out. In which, 6-gingerol as a naturally occurring plant phenol, is the most abundant component in fresh ginger. It has been shown that 6-gingerol play an anti-colorectal cancer effect by inhibiting leukotriene A (4) hydrolase expression and induction of G2/M arrest [19–21]. It is worth noting that this compound exhibited compact relationships with 17 key target genes, indicating the potential of 6-gingerol as a leading compound in colon cancer prevention. However, the LibDock Scores of 6-gingerol is lower than 1-monolinolein, which may be due to the poor DL property (DL < 0.18). Therefore, structural variation should be applied for improving its pharmacokinetics characteristics to reach a reasonable drug-like property. Other active components, like beta-sitosterol, a main dietary phytosterol in plants, was found to be preventive on the growth of HT116 human colon cancer cells and this function was associated with induction of Bax and activation of Caspases [22]. This above evidence indicates the activities of these ingredients in ginger for treatment of colon cancer, however, further clinical experiment is still needed, especially for the most active component of 1-monolinolein.

By target fishing, a total of 34 key genes were screened as the most important ones that contribute to the colon cancer prevention of ginger. These potential target genes were placed in the Metascape for KEGG pathway analysis, and 20 significant pathways that may be regulated by ginger in the treatment of colon cancer were identified, including pathways in cancer, endocrine resistance, hepatitis B, PI3K-Akt signaling pathway, and EGFR tyrosine kinase inhibitor resistance. Importantly, PI3K-Akt pathway is a critical signal cascade focusing on serine/threonine kinase Akt, and AKT1, PIK3CA, MAPK3, TP53 genes were involved in this pathway. It perhaps the most commonly activated signaling pathway in human cancer and misregulation of the PI3K-Akt pathway has been revealed to be closely associated with pathogenetic process of colon cancer [23, 24]. By analyzing GO enrichment, we found that phosphatidylinositol 3-kinase signaling is an important biological process of ginger on regulating cancer-related pathways, which may be contribute to the regulation of PI3K-Akt pathway in this study. Another signaling pathway of EGFR tyrosine kinase inhibitor have been effectively used as a promising target for clinical treatment of non-squamous non-small cell lung cancer [25, 26]. However, some of responders relapsed because of acquired resistance. Recent studies showed that combination of EGFR tyrosine kinase inhibitor and AKT inhibitors could be a rational therapeutic approach for lung cancer patients [27]. Therefore, PI3K-Akt signaling pathway and EGFR tyrosine kinase inhibitor resistance pathways can be considered as the significant pathways of ginger for treating colon cancer. Besides, some biological process, such as cellular response to oxidative stress, has been found to take part in regulating signaling pathways related to human colon cancer [28, 29]. Reactive oxygen species (ROS) is an umbrella term for an array of derivatives of oxygen, and elevated level of ROS resulted in molecular damage, denoted as “oxidative distress” [30]. It is increasingly clear that reactive oxygen species (ROS) including superoxide and hydrogen peroxide, positioned at a crossroads potentially linking the cancer and immune microenvironment [31]. Further study demonstrated that targeting mitochondrial complex I with metformin led to reduction of complex I generated ROS and reduced tumorigenesis in xenograft mouse models [32].

According to the compound-target-pathway-disease interaction network, each active component acts on at least one target genes and most of the target acts on at least one pathway. For example, TP53 as a tumor suppressor gene was found to be involved in 11 pathways, such as EGFR tyrosine kinase inhibitor resistance and PI3K-Akt signaling pathway. Another important gene of PIK3R1 was associated with all the significant pathways. Furthermore, the core targets TP53, HSP90AA1, and JAK2 were verified to be the potential targets of ginger for treating colon cancer. These findings revealed that the anti-cancer effect of ginger was played through regulating multi-targets by multi-components. However, the illustration in the molecular levels of ginger prevented colon cancer need to be provided in the future. Meanwhile, another limitation of this investigation is that it was focused on lipid substances of ginger, and large molecular compounds such as polysaccharides and proteins were not included. Further research is required to supplement this data.

## Conclusion

Collectively, 6 active components were identified from ginger and 285 targets in addition to 1356 disease-related targets in the treatment of colon cancer were collected. In the String analysis, a total of 34 candidate targets were yielded, and 10 signaling pathways including pathways in cancer, PI3K-Akt signaling pathway, and EGFR tyrosine kinase inhibitor resistance were observed to play an important role in the mechanism of ginger for colon cancer prevention. In addition, TP53, HSP90AA1, and JAK2 were speculated to be the most important target proteins. These results might be promising to searching for leading compounds and the developments of new drug for colon cancer, however, continued experiments are demanded to available the findings.

## Data Availability

All data are available in the manuscript and they are exhibited in figures and tables.
